# Metal halide coordination compounds with quinazolin-4(3*H*)-one

**DOI:** 10.1107/S2056989023004802

**Published:** 2023-06-06

**Authors:** Kambarali K. Turgunov, Ulli Englert

**Affiliations:** a S.Yunusov Institute of the Chemistry of Plant Substances, Academy of Sciences of Uzbekistan, Mirzo Ulugbek Str., 77, Tashkent 100170, Uzbekistan; b Turin Polytechnic University in Tashkent, Kichik Khalka yuli str. 17, 100095 Tashkent, Uzbekistan; c Institute of Inorganic Chemistry, RWTH Aachen University, Landoltweg 1, 52056, Aachen, Germany; Vienna University of Technology, Austria

**Keywords:** crystal structure, cadmium(II) coordination polymer, halide-bridged coordination polymers, mercury(II) coordination polymer, quinazolinone coordination, tetra­hedral cadmium(II) complex.

## Abstract

Three coordination compounds of quinazolin-4(3*H*)-one (**quinoz**; C_8_H_6_N_2_O) with divalent group 12 halides are reported. In all complexes, coordination occurs *via* the nitro­gen atom *ortho* to the quinazolinone carbonyl group. In the two chain polymers with composition [*MX*_2_(**quinoz**)], *viz*. (*M* = Cd, *X* = Br), *catena*-poly[[[quinazolin-4(3*H*)-one-κ*N* ^3^]cadmium(II)]-di-μ-bromido], [CdBr_2_(C_8_H_6_N_2_O)]_*n*_ (**I**), and *M* = Hg, *X* = Cl, *catena*-poly[[[quinazolin-4(3*H*)-one-κ*N* ^3^]mercury(II)]-di-μ-chlorido], [HgCl_2_(C_8_H_6_N_2_O)]_*n*_ (**II**), the divalent cations are five-coordinate, with four bridging halide and one terminal **quinoz** ligand. The Cd^II^ atom in (**I**) has an almost trigonal–bipyramidal coordination environment, whereas the Hg^II^ atom in (**II**) has a more distorted coordination environment. Likewise, the halide bridges in (**II**) are significantly more asymmetric than in (**I**). In both (**I**) and (**II**), **quinoz** ligands at adjacent cations along each strand are oriented in opposite directions, and the organic ligands of neighboring strands inter­digitate with resulting π–π inter­actions. In contrast to the halide-bridged chain polymers (**I**) and (**II**), the adduct of **quinoz** with CdI_2_ is the tetra­hedral complex [CdI_2_(**quinoz**)_2_], di­iodido­bis­[quinazolin-4(3*H*)-one-κ*N* ^3^]cadmium(II), [CdI_2_(C_16_H_12_N_4_O_2_)], (**III**). The Cd^II^ atom in this discrete complex is located on a twofold rotation axis. Disorder in (**III**) is reflected in an alternative minority orientation of the mol­ecules for which the iodine sites closely match the position of the majority orientation. In view of the low site occupancy of only 0.0318 (8) Å, only the Cd^II^ position for this alternative orientation was taken into account during refinement. In all three compounds, classical N—H⋯O hydrogen bonds with donor–acceptor distances of *ca* 2.9 Å occur; they link the polymer chains in (**I**) and (**II**) into di-periodic networks and connect adjacent discrete complexes in (**III**) to mono-periodic strands.

## Abstract 2

The cadmium(II) and mercury (II) coordination compounds with composition [CdBr_2_(quinoz)] and [HgCl_2_(quinoz)], respectively, where quinoz = quinazolin-4(3*H*)-one, are chain polymers with bridging halogen atoms. The structure of [CdI_2_(quinoz)_2_] was re-determined at 100 K, modeling minor disorder.

## 1. Chemical context

**Figure 1:**
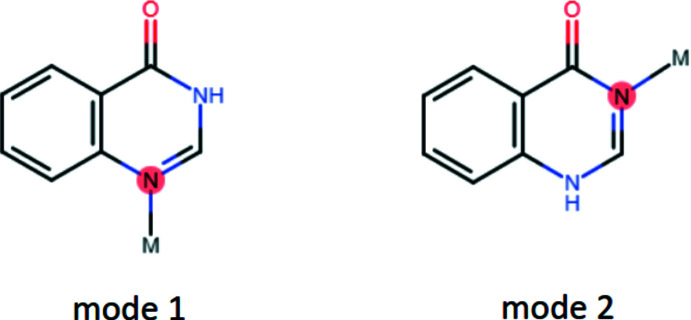
The two possible types of coordination modes for the quinazolin-4-one ligand in metal complexes.

4(3*H*)-Quinazolinone (**quinoz**) can act as ligand for metal ions in different coordination modes. Both coordination through the nitro­gen atom *para* (mode 1) and, after tautomerization, *via* the nitro­gen atom *ortho* to the quinazolinone carbonyl group (mode 2) have been observed (Fig. 1 ▸). An Ag^I^ coord­in­ation compound (Li *et al.*, 2015 ▸) provides an example for the co-existence of both binding modes in the same crystal structure. Earlier studies on the reaction products of cadmium chloride or bromide with quinazolin-4(3*H*)-one have shown that the **quinoz** ligand may inter­act with Cd^II^ cations *via* the *para* nitro­gen atom, *i.e*. according to mode 1. Four bridging halides in the equatorial plane and two **quinoz** ligands in a *trans*-axial arrangement give rise to a pseudo-octa­hedral coordination environment around the metal cation (Turgunov & Englert, 2010 ▸; Turgunov *et al.*, 2010 ▸; Shomurotova *et al.*, 2012 ▸; Đaković *et al.*, 2018 ▸). 
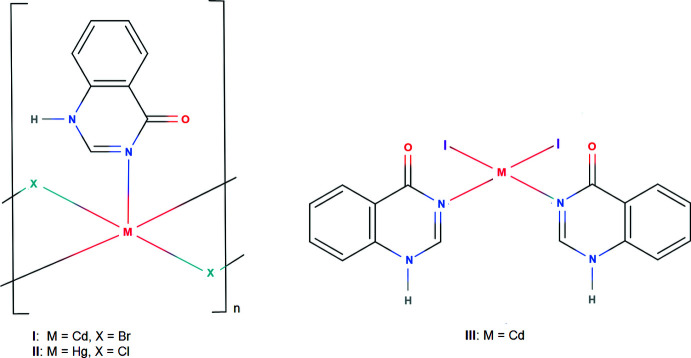


We here report three other examples for coordination according to mode 2, namely the adducts of quinoz with CdBr_2_ (**I**), HgCl_2_ (**II**) and CdI_2_ (**III**). The influence of different halide ligands on the coordination environment of divalent cations with N-donor co-ligands has been discussed in detail (Hu & Englert, 2001 ▸, 2002 ▸; Hu *et al.*, 2003 ▸).

## 2. Structural commentary

**Figure 2:**
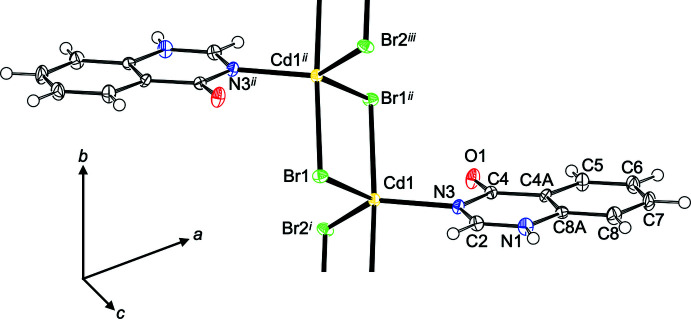
Section of polymer (**I**) with the atom-numbering scheme. Displacement ellipsoids are drawn at the 50% probability level, H atoms are shown as spheres of arbitrary radius. [Symmetry codes: (i) −*x*, 1 − *y*, 1 − *z*; ii) −*x*, 2 − *y*, 1 − *z*; iii) *x*, 1 + *y*, *z*].

**Figure 3:**
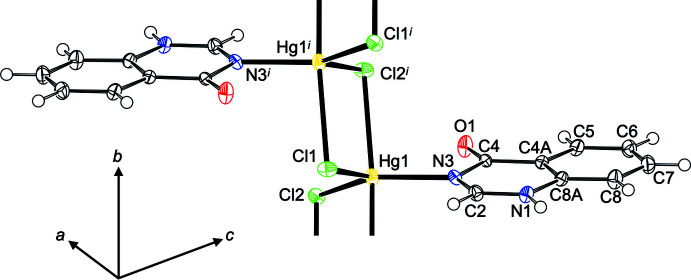
Section of polymer (**II**) with the atom-numbering scheme. Displacement ellipsoids are drawn at the 50% probability level, H atoms are shown as spheres of arbitrary radius. [Symmetry code: (i) 2 − *x*, 1 − *y*, −*z*].

**Figure 4:**
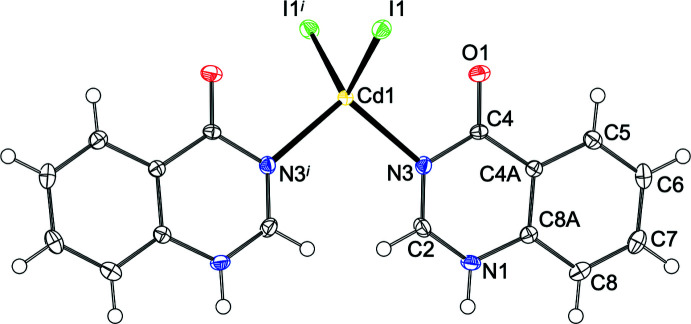
Mol­ecular structure of (**III**) with the atom-numbering scheme; the minor disorder of the Cd site is shown in Fig. 9 ▸ and has been omitted here. Displacement ellipsoids are drawn at the 50% probability level, H atoms are shown as spheres of arbitrary radius. [Symmetry code: (i) −*x*, *y*, 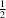 − *z*]

The asymmetric unit of (**I**) consists of a Cd^II^ cation, two Br^−^ ligands and one **quinoz** ligand attached in mode 2 (Fig. 2 ▸). The cation adopts a coordination number of 5 and is characterized by a τ_5_ descriptor (Addison *et al.*, 1984 ▸) of 0.80. In an alternative description (Holmes, 1984 ▸), its shape corresponds to only 5% distorsion along a hypothetical pathway from *D*_3*h*_ to *C*_4*v*_. Both qualifiers consistently assign this shape as trigonal bipyramidal, with the **quinoz** ligand in an equatorial position. The equatorial plane defined by Cd1, Br1, Br2^i^ [symmetry code: (i) −*x*, 1 − *y*, 1 − *z*] and N3 and the least-squares plane through the **quinoz** ligand subtend a dihedral angle of 38.32 (13)°. The bromido ligands act as rather symmetric bridges between neighboring cations, thus giving rise to a chain polymer extending along [010]. Additional details concerning the crystal structure of (**I**) are best discussed together with the related derivative (**II**) (Fig. 3 ▸). Both compounds share the same composition [*M*X_2_(**quinoz**)], with bridging halide ligands between neighboring divalent group 12 cations at a distance slightly less than 4 Å. The mercury compound (**II**) shows a considerably more distorted coordination environment than its cadmium congener (**I**): On the one hand, the coordination environment about the cation is less regular; both the τ_5_ (0.56) and the Holmes descriptor (23%) assign a shape in-between trigonal bipyramidal and square pyramidal. On the other hand, the chlorido bridges in (**II**) are significantly more asymmetric than the bromido linkers in (**I**). Even more asymmetric halide bridges have been observed in the bis adduct of 1,2,3,9-tetra­hydro-pyrrolo­[2,1-*b*]quinazolin-9-one to HgCl_2_ (Turgunov *et al.*, 2011 ▸). Both chain polymers (**I**) and (**II**) fit well into the wider context of halide-bridged chain polymers. The adducts of donor ligands to CdBr_2_ or HgCl_2_ mostly display coordination numbers of 5 or 6 and have bridging halide ligands. For such bromido-bridged Cd^II^ strands, similar Cd–Cd separations as in (**I**) [Cd1⋯Cd1^i^ = 3.8667 (10) and Cd1⋯Cd1^ii^ = 3.9051 (10) Å; symmetry codes: (i) −*x*, 2 − *y*, 1 − *z*; (ii) −*x*, 1 − *y*, 1 − *z*] have been reported (Hu & Englert, 2002 ▸; Merkens *et al.*, 2014 ▸; Hu *et al.*, 2003 ▸). The Hg–Hg separations [Hg1⋯Hg1^i^ = 3.7881 (6) and Hg1⋯Hg1^ii^ = 3.8827 (6) Å, symmetry codes: (i) 2 − *x*, 1 − *y*, −*z*; (ii) 2 − *x*, −*y*, −*z*] in (**II**) are comparable to those encountered in related chlorido-bridged polymers (Hu *et al.*, 2007 ▸; Truong *et al.*, 2017 ▸; van Terwingen *et al.*, 2021 ▸; Merkens *et al.*, 2010 ▸). A different situation arises for (**III**) (Fig. 4 ▸): for the bis­(ligand) adduct of CdI_2_, a discrete complex may be expected and is indeed encountered. The CSD database (Groom *et al.*, 2016 ▸) contains only a few structures for six-coordinated Cd with four iodido and two additional arbitrary ligands, for example a di-periodic structure with bipyridyl ligands in one and iodido bridges in a second direction (Hu *et al.*, 2003 ▸). In contrast, more than 600 hits for tetra­hedrally coordinated Cd^II^ with two iodido and two additional ligands have been documented, and (**III**) falls into this category. The crystal structure of (**III**) has been previously reported by Đaković *et al.* (2018 ▸). Our present report takes a minor disorder into account, which explains an otherwise unaccounted high residual electron density; details are provided in the *Refinement* section. In (**III**), the cation resides on a twofold rotation axis of space group *C*2/*c*, Wyckoff position 4*e*. Its coordination environment is characterized by a τ_4_ descriptor (Yang *et al.*, 2007 ▸) of 0.93, corresponding to an almost undistorted tetra­hedron.

## 3. Supra­molecular features

**Figure 5:**
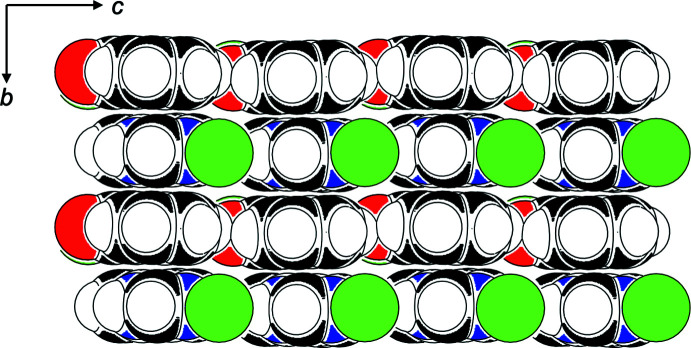
Space-filling model for (**I**) (*PLUTO*; Spek 2009 ▸) as viewed along [100]; Br atoms have been omitted. Color code: Cd green, C black, O red, N blue, H white.

**Figure 6:**
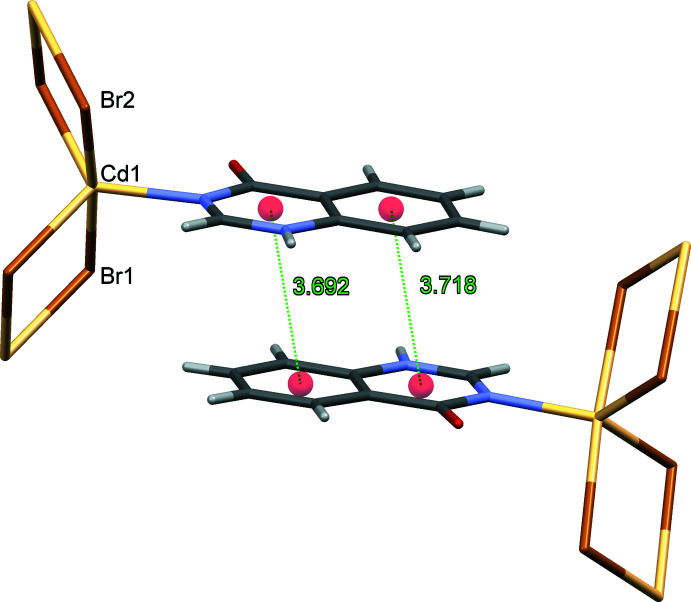
The relevant π–π inter­actions in the crystal structure of (**I**).

Classical N—H⋯O hydrogen bonds exist in structures (**I**)–(**III**). They link the NH group to the carbonyl oxygen atom of a neighboring **quinoz** ligand [parallel to [001] for (**I**) and (**II**), and parallel to [010] for (**III**)], and involve donor–acceptor distances around 2.9 Å. Numerical details of the hydrogen-bonding inter­actions are compiled in Tables 1 ▸–3 ▸ ▸. In the coordination polymers (**I**) and (**II**), **quinoz** ligands of adjacent strands inter­digitate. The distances between neighboring coplanar organic ligands amount to one half of the lattice parameter *b*, *i.e*. 3.5–3.6 Å and suggest π–π stacking. As an example, a space-filling model for (**I**) (Fig. 5 ▸) shows the close approach between organic **quinoz** ligands on neighboring strands. An analysis with *PLATON* (Spek, 2020 ▸) gives numerical values of π–π stacking inter­actions observed between two parallel **quinoz** ligands for crystals of (**I**)–(**III**)**:** *Cg*(pyrimidine ring)⋯*Cg*(benzene ring) distances are 3.6923 (3) Å (slippage 0.843 Å) and 3.718 (3) Å (0.906 Å) in (**I**), 3.7042 (4) Å (1.003 Å) in (**II**) and 3.5578 (14) Å (1.185 Å) in (**III**) (Figs. 6 ▸–8 ▸ ▸).

## 4. Synthesis and crystallization

Compound (**I**). 70 mg (0.2 mmol) of cadmium bromide tetra­hydrate were dissolved in a mixture of 4 ml of ethanol and 1 ml of water. 60 mg (0.4 mmol) of quinazolin-4(3*H*)-one dissolved in 5 ml of ethanol were added to the cadmium bromide solution. Crystals started to precipitate after a few minutes, and colorless prismatic crystals suitable for single-crystal X-ray diffraction analysis formed within 2–3 h.

Compound (**II**). 54.3 mg (0.2 mmol) of HgCl_2_ were dissolved in ∼3 ml acetone. 30 mg (0.2 mmol) of quinazolin-4(3*H*)-one were dissolved in 3 ml of acetone under mild heating, and the resulting solution was added to the HgCl_2_ solution. Colorless prismatic crystals suitable for X-ray diffraction analysis formed within seconds.

Compound (**III**): 73 mg (0.2 mmol) of CdI_2_ were dissolved in 1 ml of ethanol. 60 mg (0.4 mmol) of the ligand were dissolved in 4 ml of ethanol under mild heating, and the resulting solution was added to the CdI_2_ solution. After slow evaporation of the solvent at ambient temperature for several days, colorless single crystals suitable for X-ray diffraction analysis were obtained.

## 5. Refinement

Crystal data, data collection and structure refinement details are summarized in Table 4 ▸. Positional parameters for H atoms attached to N atoms were refined, H atoms bonded to carbon were introduced in calculated positions and treated as riding on their parent atoms.

**Figure 9:**
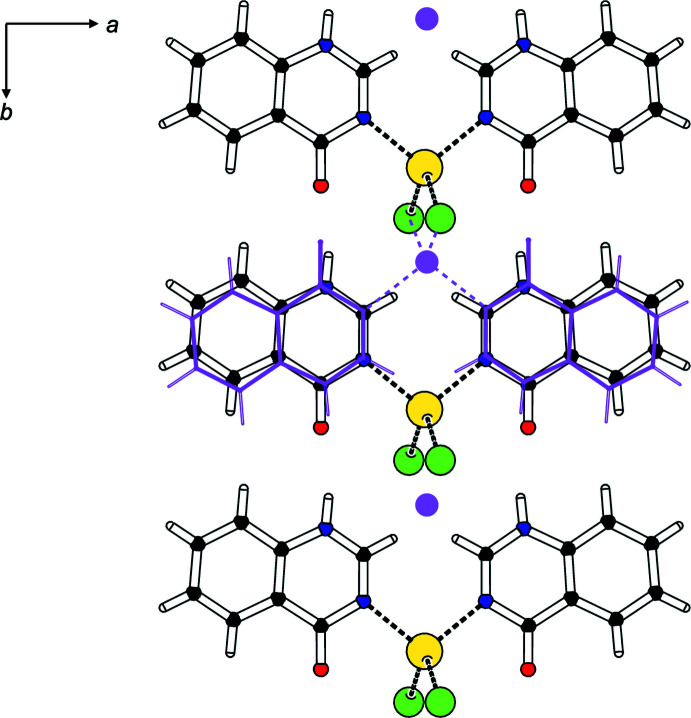
Disorder in (**III**). The alternative Cd site (Cd2) is shown as a magenta-colored sphere. For clarity, the alternative ligand orientations are also shown in magenta. However, they have not been revealed experimentally and were not taken into account during refinement.

Several crystals of (**I**) were tested and proved to be twinned; two domains of roughly equal volume are related by a 180° rotation about the *c* axis. The specimen selected for intensity data collection showed *ca* 12000 overlapped out of a total of 65000 reflections. Final refined component fractions amounted to 0.5569 (8):0.4431 (8). Crystals of (**II**) were also twinned by non-merohedry. Here, two domains of roughly equal volume are related by a 180° rotation about the *b* axis. In the selected crystal, two domains contributed to *ca* 2000 overlapped out of a total of *ca* 14000 reflections. Final refined component fractions amounted to 0.5178 (9):0.4822 (9). The crystal selected for intensity data collection for (**III**) was a single crystal. After completion of the structure model, a difference-Fourier map showed a local density maximum of *ca* 5 electrons/ Å^3^ not associated with any atom site. This position subtended distances to the iodine atoms similar to Cd1—I1. We suggest that this residual electron density represents an alternative Cd site. In the final refinement, the sum of the site occupancies for the positionally disordered Cd sites was constrained to unity, and both sites were constrained to share the same anisotropic displacement parameters. Fig. 9 ▸ explains the arrangement of the mol­ecules in both alternative orientations; the minority orientation is depicted in magenta. As the minority Cd site refined to an occupancy of only 0.0318 (8) and the iodine ligands for both orientations closely overlap, no attempt was made to detect and refine the alternative sites for the light atoms associated with the **quinoz** ligand. Inter­estingly, the authors of the previous crystal-structure determination of (**III**) (Đaković *et al.*, 2018 ▸) encountered the same local density maximum (but without modeling the disorder). Hence, the disorder appears to be a feature of the crystal structure and not of the individual crystal chosen for the data collection.

## Supplementary Material

Crystal structure: contains datablock(s) I, II, III, GLOBAL. DOI: 10.1107/S2056989023004802/wm5681sup1.cif

Structure factors: contains datablock(s) I. DOI: 10.1107/S2056989023004802/wm5681Isup5.hkl

Structure factors: contains datablock(s) II. DOI: 10.1107/S2056989023004802/wm5681IIsup6.hkl

Structure factors: contains datablock(s) III. DOI: 10.1107/S2056989023004802/wm5681IIIsup7.hkl

CCDC references: 2267121, 2267120, 2267119

Additional supporting information:  crystallographic information; 3D view; checkCIF report

## Figures and Tables

**Figure 7:**
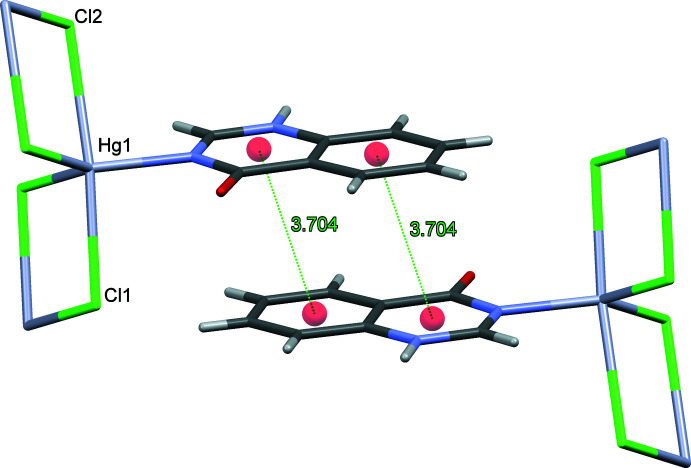
The relevant π–π inter­actions in the crystal structure of (**II**).

**Figure 8:**
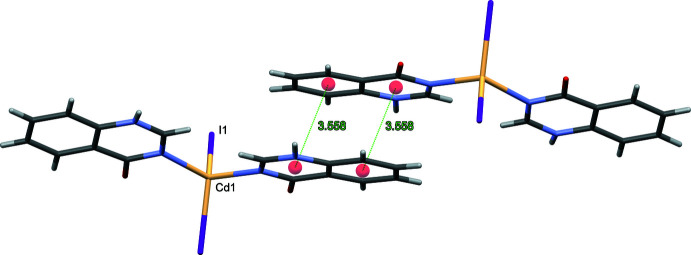
The relevant π–π inter­actions in the crystal structure of (**III**).

**Table 1:** Hydrogen-bond geometry (Å, °) for (**I**)

*D*—H⋯*A*	*D*—H	H⋯*A*	*D*⋯*A*	*D*—H⋯*A*
N1—H1⋯O1^i^	0.87 (3)	2.10 (3)	2.917 (6)	155 (6)

Symmetry code: (i) 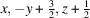 .

**Table 2:** Hydrogen-bond geometry (Å, °) for (**II**)

*D*—H⋯*A*	*D*—H	H⋯*A*	*D*⋯*A*	*D*—H⋯*A*
N1—H1⋯O1^i^	0.89 (4)	2.11 (4)	2.935 (8)	155 (7)

Symmetry code: (i) 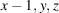 .

**Table 3:** Hydrogen-bond geometry (Å, °) for (**III**)

*D*—H⋯*A*	*D*—H	H⋯*A*	*D*⋯*A*	*D*—H⋯*A*
N1—H1*A*⋯O1^i^	0.93 (3)	1.97 (3)	2.893 (3)	168 (4)

Symmetry code: (i) 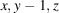 .

**Table 4:** Experimental details

	(**I**)	(**II**)	(**III**)
Crystal data			
Chemical formula	[CdBr_2_(C_8_H_6_N_2_O)]	[HgCl_2_(C_8_H_6_N_2_O)]	[CdI_2_(C_16_H_12_N_4_O_2_)]
*M*_r_	418.37	417.64	658.50
Crystal system, space group	Monoclinic, *P*2_1_/*c*	Triclinic, *P* 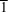	Monoclinic, *C*2/*c*
Temperature (K)	100	100	100
*a*, *b*, *c* (Å)	10.7930 (11), 7.2019 (7), 13.7605 (14)	6.8191 (8), 7.0735 (8), 10.4659 (12)	22.242 (3), 6.8450 (9), 13.3702 (17)
α, β, γ (°)	90, 100.4705 (18), 90	85.718 (2), 80.7887 (19), 89.152 (2)	90, 118.8220 (16), 90
*V* (Å^3^)	1051.79 (18)	496.92 (10)	1783.4 (4)
*Z*	4	2	4
Radiation type	Mo *K*α	Mo *K*α	Mo *K*α
μ (mm^−1^)	9.64	15.99	4.70
Crystal size (mm)	0.25 × 0.10 × 0.05	0.04 × 0.03 × 0.03	0.12 × 0.10 × 0.04

Data collection			
Diffractometer	Bruker D8 gonimeter with APEX CCD detector	Bruker D8 gonimeter with APEX CCD detector	Bruker D8 gonimeter with APEX CCD detector
Absorption correction	Multi-scan (*TWINABS*; Bruker, 2014 ▸)	Multi-scan (*TWINABS*; Bruker, 2014 ▸)	Multi-scan (*SADABS*; Bruker, 2014 ▸)
*T*_min_, *T*_max_	0.446, 0.746	0.302, 0.433	0.544, 0.746
No. of measured, independent and observed [*I* > 2σ(*I*)] reflections	65521, 5868, 4717	14434, 5037, 4693	13210, 2687, 2519
*R*_int_	0.089	0.050	0.024
(sin θ/λ)_max_ (Å^−1^)	0.718	0.709	0.717

Refinement			
*R*[*F* ^2^ > 2σ(*F* ^2^)], *wR*(*F* ^2^), *S*	0.039, 0.076, 1.04	0.036, 0.076, 1.08	0.026, 0.063, 1.12
No. of reflections	5868	5037	2687
No. of parameters	132	132	119
No. of restraints	1	1	2
H-atom treatment	H atoms treated by a mixture of independent and constrained refinement	H atoms treated by a mixture of independent and constrained refinement	H atoms treated by a mixture of independent and constrained refinement
Δρ_max_, Δρ_min_ (e Å^−3^)	1.13, −0.96	1.54, −1.49	3.17, −0.50

Computer programs: *APEX2* and *SAINT* (Bruker, 2014 ▸), *SHELXT* (Sheldrick, 2015*a* ▸), *SHELXL* (Sheldrick, 2015*b* ▸), *PLATON* (Spek, 2020 ▸) and *publCIF* (Westrip, 2010 ▸).

## supplementary crystallographic information

### catena-Poly[[[quinazolin-4(3H)-one-κN3]cadmium(II)]-di-µ-bromido] (I) Crystal data

**Table:**

[CdBr_2_(C_8_H_6_N_2_O)]	*F*(000) = 776
*M**_r_* = 418.37	*D*_x_ = 2.642 Mg m^−^^3^
Monoclinic, *P*2_1_/*c*	Mo *K*α radiation, λ = 0.71073 Å
*a* = 10.7930 (11) Å	Cell parameters from 3537 reflections
*b* = 7.2019 (7) Å	θ = 3.0–26.0°
*c* = 13.7605 (14) Å	µ = 9.64 mm^−^^1^
β = 100.4705 (18)°	*T* = 100 K
*V* = 1051.79 (18) Å^3^	Rod, colourless
*Z* = 4	0.25 × 0.10 × 0.05 mm

### catena-Poly[[[quinazolin-4(3H)-one-κN3]cadmium(II)]-di-µ-bromido] (I) Data collection

**Table:**

Bruker D8 gonimeter with APEX CCD detector diffractometer	5868 independent reflections
Radiation source: Incoatec microsource	4717 reflections with *I* > 2σ(*I*)
Multilayer optics monochromator	*R*_int_ = 0.089
ω scans	θ_max_ = 30.7°, θ_min_ = 3.0°
Absorption correction: multi-scan (*TWINABS*; Bruker, 2014)	*h* = −15→14
*T*_min_ = 0.446, *T*_max_ = 0.746	*k* = 0→10
65521 measured reflections	*l* = 0→19

### catena-Poly[[[quinazolin-4(3H)-one-κN3]cadmium(II)]-di-µ-bromido] (I) Refinement

**Table:**

Refinement on *F*^2^	Primary atom site location: structure-invariant direct methods
Least-squares matrix: full	Hydrogen site location: mixed
*R*[*F*^2^ > 2σ(*F*^2^)] = 0.039	H atoms treated by a mixture of independent and constrained refinement
*wR*(*F*^2^) = 0.076	*w* = 1/[σ^2^(*F*_o_^2^) + (0.026*P*)^2^ + 1.8*P*] where *P* = (*F*_o_^2^ + 2*F*_c_^2^)/3
*S* = 1.04	(Δ/σ)_max_ = 0.002
5868 reflections	Δρ_max_ = 1.13 e Å^−^^3^
132 parameters	Δρ_min_ = −0.96 e Å^−^^3^
1 restraint	

### catena-Poly[[[quinazolin-4(3H)-one-κN3]cadmium(II)]-di-µ-bromido] (I) Special details

**Table:**

Geometry. All esds (except the esd in the dihedral angle between two l.s. planes) are estimated using the full covariance matrix. The cell esds are taken into account individually in the estimation of esds in distances, angles and torsion angles; correlations between esds in cell parameters are only used when they are defined by crystal symmetry. An approximate (isotropic) treatment of cell esds is used for estimating esds involving l.s. planes.
Refinement. Refined as a 2-component twin.

### catena-Poly[[[quinazolin-4(3H)-one-κN3]cadmium(II)]-di-µ-bromido] (I) Fractional atomic coordinates and isotropic or equivalent isotropic displacement parameters (Å^2^)

**Table:**

	*x*	*y*	*z*	*U*_iso_*/*U*_eq_	
Cd1	0.06536 (3)	0.75144 (5)	0.52545 (2)	0.01158 (8)	
Br1	−0.12368 (4)	0.94278 (7)	0.57455 (4)	0.01378 (11)	
Br2	−0.01900 (5)	0.45101 (7)	0.63243 (4)	0.01525 (11)	
O1	0.3587 (3)	0.7297 (5)	0.5233 (3)	0.0182 (8)	
N1	0.3312 (4)	0.7386 (6)	0.8091 (3)	0.0145 (8)	
H1	0.315 (6)	0.738 (8)	0.869 (2)	0.029 (17)*	
C2	0.2323 (4)	0.7393 (7)	0.7372 (4)	0.0138 (9)	
H2	0.151110	0.739229	0.754639	0.017*	
N3	0.2391 (4)	0.7401 (6)	0.6423 (3)	0.0122 (8)	
C4	0.3551 (4)	0.7349 (7)	0.6125 (4)	0.0126 (9)	
C4A	0.4672 (4)	0.7350 (7)	0.6901 (4)	0.0117 (9)	
C5	0.5890 (5)	0.7309 (7)	0.6692 (4)	0.0175 (10)	
H5	0.600677	0.726430	0.602429	0.021*	
C6	0.6918 (5)	0.7333 (8)	0.7441 (4)	0.0197 (11)	
H6	0.774211	0.731509	0.728953	0.024*	
C7	0.6761 (5)	0.7385 (8)	0.8425 (4)	0.0199 (11)	
H7	0.747907	0.741545	0.893885	0.024*	
C8	0.5571 (5)	0.7391 (8)	0.8656 (4)	0.0187 (11)	
H8	0.546255	0.739904	0.932618	0.022*	
C8A	0.4523 (4)	0.7384 (7)	0.7886 (3)	0.0122 (9)	

### catena-Poly[[[quinazolin-4(3H)-one-κN3]cadmium(II)]-di-µ-bromido] (I) Atomic displacement parameters (Å^2^)

**Table:**

	*U*^11^	*U*^22^	*U*^33^	*U*^12^	*U*^13^	*U*^23^
Cd1	0.01016 (15)	0.01311 (15)	0.01124 (16)	0.00003 (14)	0.00135 (12)	0.00022 (13)
Br1	0.0145 (2)	0.0135 (2)	0.0149 (2)	0.00045 (19)	0.00676 (19)	0.00145 (19)
Br2	0.0203 (2)	0.0149 (2)	0.0113 (2)	−0.0055 (2)	0.00477 (19)	−0.00073 (19)
O1	0.0152 (17)	0.031 (2)	0.0085 (17)	0.0037 (16)	0.0025 (14)	0.0009 (15)
N1	0.0140 (19)	0.024 (2)	0.0074 (19)	0.0003 (18)	0.0071 (16)	−0.0001 (19)
C2	0.012 (2)	0.016 (2)	0.014 (2)	0.001 (2)	0.0053 (18)	−0.001 (2)
N3	0.0101 (18)	0.0153 (19)	0.011 (2)	0.0016 (17)	0.0014 (15)	0.0012 (17)
C4	0.011 (2)	0.013 (2)	0.014 (2)	−0.0006 (19)	0.0013 (18)	0.002 (2)
C4A	0.010 (2)	0.013 (2)	0.011 (2)	0.0027 (18)	0.0011 (18)	0.003 (2)
C5	0.013 (2)	0.028 (3)	0.012 (2)	−0.001 (2)	0.003 (2)	0.003 (2)
C6	0.010 (2)	0.028 (3)	0.021 (3)	−0.002 (2)	0.0029 (19)	0.005 (2)
C7	0.016 (2)	0.030 (3)	0.012 (2)	−0.002 (2)	−0.0035 (19)	0.004 (2)
C8	0.019 (3)	0.027 (3)	0.009 (2)	−0.002 (2)	0.0016 (19)	0.002 (2)
C8A	0.012 (2)	0.015 (2)	0.010 (2)	−0.0025 (19)	0.0021 (18)	0.004 (2)

### catena-Poly[[[quinazolin-4(3H)-one-κN3]cadmium(II)]-di-µ-bromido] (I) Geometric parameters (Å, º)

**Table:**

Cd1—N3	2.238 (4)	C4—C4A	1.460 (6)
Cd1—Br2^i^	2.5886 (6)	C4A—C8A	1.394 (6)
Cd1—Br1	2.6494 (6)	C4A—C5	1.396 (6)
Cd1—Br1^ii^	2.7302 (6)	C5—C6	1.371 (7)
Cd1—Br2	2.8574 (6)	C5—H5	0.9500
O1—C4	1.236 (5)	C6—C7	1.395 (7)
N1—C2	1.317 (6)	C6—H6	0.9500
N1—C8A	1.386 (6)	C7—C8	1.378 (7)
N1—H1	0.88 (2)	C7—H7	0.9500
C2—N3	1.321 (6)	C8—C8A	1.403 (7)
C2—H2	0.9500	C8—H8	0.9500
N3—C4	1.388 (6)		
			
N3—Cd1—Br2^i^	126.23 (10)	O1—C4—N3	119.2 (4)
N3—Cd1—Br1	114.78 (10)	O1—C4—C4A	123.7 (4)
Br2^i^—Cd1—Br1	117.82 (2)	N3—C4—C4A	117.1 (4)
N3—Cd1—Br1^ii^	98.72 (11)	C8A—C4A—C5	118.7 (4)
Br2^i^—Cd1—Br1^ii^	93.35 (2)	C8A—C4A—C4	119.0 (4)
Br1—Cd1—Br1^ii^	88.152 (19)	C5—C4A—C4	122.3 (4)
N3—Cd1—Br2	84.61 (11)	C6—C5—C4A	120.6 (5)
Br2^i^—Cd1—Br2	88.56 (2)	C6—C5—H5	119.7
Br1—Cd1—Br2	85.999 (18)	C4A—C5—H5	119.7
Br1^ii^—Cd1—Br2	174.08 (2)	C5—C6—C7	120.4 (5)
Cd1—Br1—Cd1^ii^	91.846 (19)	C5—C6—H6	119.8
Cd1^i^—Br2—Cd1	91.44 (2)	C7—C6—H6	119.8
C2—N1—C8A	120.8 (4)	C8—C7—C6	120.5 (5)
C2—N1—H1	116 (4)	C8—C7—H7	119.8
C8A—N1—H1	124 (4)	C6—C7—H7	119.8
N1—C2—N3	124.1 (4)	C7—C8—C8A	118.9 (5)
N1—C2—H2	118.0	C7—C8—H8	120.6
N3—C2—H2	118.0	C8A—C8—H8	120.6
C2—N3—C4	120.4 (4)	N1—C8A—C4A	118.5 (4)
C2—N3—Cd1	121.4 (3)	N1—C8A—C8	120.4 (4)
C4—N3—Cd1	118.2 (3)	C4A—C8A—C8	121.0 (4)
			
C8A—N1—C2—N3	−0.1 (8)	C4—C4A—C5—C6	179.2 (5)
N1—C2—N3—C4	1.8 (8)	C4A—C5—C6—C7	0.5 (8)
N1—C2—N3—Cd1	−177.8 (4)	C5—C6—C7—C8	0.7 (9)
C2—N3—C4—O1	177.7 (5)	C6—C7—C8—C8A	−1.3 (8)
Cd1—N3—C4—O1	−2.7 (6)	C2—N1—C8A—C4A	−1.3 (7)
C2—N3—C4—C4A	−2.0 (7)	C2—N1—C8A—C8	179.5 (5)
Cd1—N3—C4—C4A	177.7 (3)	C5—C4A—C8A—N1	−178.7 (5)
O1—C4—C4A—C8A	−179.1 (5)	C4—C4A—C8A—N1	1.1 (7)
N3—C4—C4A—C8A	0.6 (7)	C5—C4A—C8A—C8	0.4 (8)
O1—C4—C4A—C5	0.7 (8)	C4—C4A—C8A—C8	−179.8 (5)
N3—C4—C4A—C5	−179.7 (5)	C7—C8—C8A—N1	179.9 (5)
C8A—C4A—C5—C6	−1.0 (8)	C7—C8—C8A—C4A	0.8 (8)

Symmetry codes: (i) −*x*, −*y*+1, −*z*+1; (ii) −*x*, −*y*+2, −*z*+1.

### catena-Poly[[[quinazolin-4(3H)-one-κN3]cadmium(II)]-di-µ-bromido] (I) Hydrogen-bond geometry (Å, º)

**Table:**

*D*—H···*A*	*D*—H	H···*A*	*D*···*A*	*D*—H···*A*
N1—H1···O1^iii^	0.87 (3)	2.10 (3)	2.917 (6)	155 (6)

Symmetry code: (iii) *x*, −*y*+3/2, *z*+1/2.

### catena-Poly[[[quinazolin-4(3H)-one-κN3]mercury(II)]-di-µ-chlorido] (II) Crystal data

**Table:**

[HgCl_2_(C_8_H_6_N_2_O)]	*Z* = 2
*M**_r_* = 417.64	*F*(000) = 380
Triclinic, *P*1	*D*_x_ = 2.791 Mg m^−^^3^
*a* = 6.8191 (8) Å	Mo *K*α radiation, λ = 0.71073 Å
*b* = 7.0735 (8) Å	Cell parameters from 3121 reflections
*c* = 10.4659 (12) Å	θ = 3.0–29.9°
α = 85.718 (2)°	µ = 15.99 mm^−^^1^
β = 80.7887 (19)°	*T* = 100 K
γ = 89.152 (2)°	Prism, colourless
*V* = 496.92 (10) Å^3^	0.04 × 0.03 × 0.03 mm

### catena-Poly[[[quinazolin-4(3H)-one-κN3]mercury(II)]-di-µ-chlorido] (II) Data collection

**Table:**

Bruker D8 gonimeter with APEX CCD detector diffractometer	5037 independent reflections
Radiation source: Incoatec microsource	4693 reflections with *I* > 2σ(*I*)
Multilayer optics monochromator	*R*_int_ = 0.050
ω scans	θ_max_ = 30.3°, θ_min_ = 2.9°
Absorption correction: multi-scan (*TWINABS*; Bruker, 2014)	*h* = −9→9
*T*_min_ = 0.302, *T*_max_ = 0.433	*k* = −9→10
14434 measured reflections	*l* = 0→14

### catena-Poly[[[quinazolin-4(3H)-one-κN3]mercury(II)]-di-µ-chlorido] (II) Refinement

**Table:**

Refinement on *F*^2^	Primary atom site location: structure-invariant direct methods
Least-squares matrix: full	Hydrogen site location: mixed
*R*[*F*^2^ > 2σ(*F*^2^)] = 0.036	H atoms treated by a mixture of independent and constrained refinement
*wR*(*F*^2^) = 0.076	*w* = 1/[σ^2^(*F*_o_^2^) + (0.020*P*)^2^ + 2.5*P*] where *P* = (*F*_o_^2^ + 2*F*_c_^2^)/3
*S* = 1.08	(Δ/σ)_max_ < 0.001
5037 reflections	Δρ_max_ = 1.54 e Å^−^^3^
132 parameters	Δρ_min_ = −1.49 e Å^−^^3^
1 restraint	

### catena-Poly[[[quinazolin-4(3H)-one-κN3]mercury(II)]-di-µ-chlorido] (II) Special details

**Table:**

Geometry. All esds (except the esd in the dihedral angle between two l.s. planes) are estimated using the full covariance matrix. The cell esds are taken into account individually in the estimation of esds in distances, angles and torsion angles; correlations between esds in cell parameters are only used when they are defined by crystal symmetry. An approximate (isotropic) treatment of cell esds is used for estimating esds involving l.s. planes.
Refinement. Refined as a 2-component twin.

### catena-Poly[[[quinazolin-4(3H)-one-κN3]mercury(II)]-di-µ-chlorido] (II) Fractional atomic coordinates and isotropic or equivalent isotropic displacement parameters (Å^2^)

**Table:**

	*x*	*y*	*z*	*U*_iso_*/*U*_eq_	
Hg1	0.96071 (4)	0.24641 (4)	0.07042 (3)	0.01548 (9)	
Cl2	1.2589 (3)	0.0702 (3)	0.01773 (19)	0.0173 (4)	
Cl1	0.8430 (3)	0.4314 (2)	−0.1209 (2)	0.0188 (4)	
O1	0.9528 (7)	0.2176 (8)	0.3605 (5)	0.0212 (11)	
N1	0.3826 (9)	0.2857 (9)	0.3209 (6)	0.0138 (12)	
H1	0.255 (5)	0.293 (12)	0.313 (8)	0.03 (2)*	
C2	0.5279 (11)	0.2835 (10)	0.2211 (7)	0.0161 (15)	
H2	0.494018	0.298894	0.136314	0.019*	
N3	0.7156 (9)	0.2615 (8)	0.2317 (6)	0.0129 (12)	
C4	0.7779 (10)	0.2372 (10)	0.3539 (7)	0.0113 (13)	
C4A	0.6195 (11)	0.2370 (10)	0.4663 (7)	0.0134 (14)	
C5	0.6608 (11)	0.2103 (11)	0.5938 (7)	0.0161 (15)	
H5	0.793612	0.189228	0.608450	0.019*	
C6	0.5093 (12)	0.2146 (11)	0.6969 (8)	0.0203 (16)	
H6	0.537995	0.197419	0.782925	0.024*	
C7	0.3144 (12)	0.2440 (11)	0.6770 (8)	0.0202 (16)	
H7	0.211185	0.246517	0.749509	0.024*	
C8	0.2688 (11)	0.2694 (11)	0.5539 (7)	0.0193 (16)	
H8	0.135319	0.290262	0.540841	0.023*	
C8A	0.4223 (10)	0.2640 (10)	0.4475 (7)	0.0130 (14)	

### catena-Poly[[[quinazolin-4(3H)-one-κN3]mercury(II)]-di-µ-chlorido] (II) Atomic displacement parameters (Å^2^)

**Table:**

	*U*^11^	*U*^22^	*U*^33^	*U*^12^	*U*^13^	*U*^23^
Hg1	0.01190 (13)	0.02113 (15)	0.01333 (14)	0.00139 (11)	−0.00131 (9)	−0.00250 (11)
Cl2	0.0123 (9)	0.0204 (9)	0.0201 (10)	0.0022 (7)	−0.0039 (7)	−0.0058 (7)
Cl1	0.0219 (10)	0.0179 (8)	0.0194 (10)	−0.0013 (7)	−0.0120 (8)	−0.0003 (7)
O1	0.013 (3)	0.034 (3)	0.015 (3)	0.002 (2)	−0.003 (2)	0.002 (3)
N1	0.007 (3)	0.022 (3)	0.013 (3)	−0.001 (2)	−0.005 (2)	0.001 (2)
C2	0.014 (4)	0.019 (4)	0.015 (4)	0.001 (3)	−0.002 (3)	−0.003 (3)
N3	0.014 (3)	0.015 (3)	0.011 (3)	0.003 (2)	−0.003 (2)	−0.003 (2)
C4	0.013 (3)	0.013 (3)	0.008 (3)	−0.001 (3)	−0.003 (3)	−0.001 (3)
C4A	0.014 (3)	0.014 (3)	0.012 (3)	−0.002 (3)	−0.001 (3)	−0.001 (3)
C5	0.013 (3)	0.023 (4)	0.013 (4)	−0.004 (3)	−0.004 (3)	0.001 (3)
C6	0.023 (4)	0.025 (4)	0.012 (4)	−0.004 (3)	−0.002 (3)	−0.001 (3)
C7	0.018 (4)	0.025 (4)	0.016 (4)	0.000 (3)	0.002 (3)	−0.003 (3)
C8	0.014 (4)	0.028 (4)	0.016 (4)	0.000 (3)	−0.003 (3)	0.000 (3)
C8A	0.010 (3)	0.015 (3)	0.014 (3)	0.000 (3)	−0.002 (3)	0.000 (3)

### catena-Poly[[[quinazolin-4(3H)-one-κN3]mercury(II)]-di-µ-chlorido] (II) Geometric parameters (Å, º)

**Table:**

Hg1—N3	2.185 (6)	C4—C4A	1.464 (10)
Hg1—Cl2	2.3791 (18)	C4A—C8A	1.398 (10)
Hg1—Cl1	2.5416 (19)	C4A—C5	1.406 (10)
Hg1—Cl1^i^	2.7861 (18)	C5—C6	1.372 (10)
O1—C4	1.211 (8)	C5—H5	0.9500
N1—C2	1.320 (9)	C6—C7	1.389 (11)
N1—C8A	1.392 (9)	C6—H6	0.9500
N1—H1	0.89 (3)	C7—C8	1.371 (10)
C2—N3	1.309 (9)	C7—H7	0.9500
C2—H2	0.9500	C8—C8A	1.403 (10)
N3—C4	1.409 (9)	C8—H8	0.9500
			
N3—Hg1—Cl2	138.49 (16)	C8A—C4A—C5	118.8 (7)
N3—Hg1—Cl1	105.30 (16)	C8A—C4A—C4	119.7 (7)
Cl2—Hg1—Cl1	114.98 (7)	C5—C4A—C4	121.4 (7)
N3—Hg1—Cl1^i^	96.08 (16)	C6—C5—C4A	119.9 (7)
Cl2—Hg1—Cl1^i^	93.94 (6)	C6—C5—H5	120.0
Cl1—Hg1—Cl1^i^	89.50 (6)	C4A—C5—H5	120.0
Hg1—Cl1—Hg1^i^	90.50 (6)	C5—C6—C7	120.7 (7)
C2—N1—C8A	120.8 (6)	C5—C6—H6	119.7
C2—N1—H1	123 (6)	C7—C6—H6	119.7
C8A—N1—H1	116 (6)	C8—C7—C6	120.9 (7)
N3—C2—N1	124.0 (7)	C8—C7—H7	119.6
N3—C2—H2	118.0	C6—C7—H7	119.6
N1—C2—H2	118.0	C7—C8—C8A	119.0 (7)
C2—N3—C4	121.5 (6)	C7—C8—H8	120.5
C2—N3—Hg1	125.6 (5)	C8A—C8—H8	120.5
C4—N3—Hg1	112.9 (4)	N1—C8A—C4A	118.3 (6)
O1—C4—N3	119.9 (6)	N1—C8A—C8	121.0 (7)
O1—C4—C4A	124.5 (6)	C4A—C8A—C8	120.6 (7)
N3—C4—C4A	115.6 (6)		
			
C8A—N1—C2—N3	0.0 (11)	C4—C4A—C5—C6	178.7 (7)
N1—C2—N3—C4	0.2 (11)	C4A—C5—C6—C7	0.4 (12)
N1—C2—N3—Hg1	177.6 (5)	C5—C6—C7—C8	0.0 (12)
C2—N3—C4—O1	−179.8 (7)	C6—C7—C8—C8A	0.4 (12)
Hg1—N3—C4—O1	2.4 (8)	C2—N1—C8A—C4A	−1.0 (10)
C2—N3—C4—C4A	0.5 (10)	C2—N1—C8A—C8	178.9 (7)
Hg1—N3—C4—C4A	−177.3 (5)	C5—C4A—C8A—N1	−178.5 (6)
O1—C4—C4A—C8A	178.9 (7)	C4—C4A—C8A—N1	1.6 (10)
N3—C4—C4A—C8A	−1.4 (9)	C5—C4A—C8A—C8	1.6 (10)
O1—C4—C4A—C5	−1.0 (11)	C4—C4A—C8A—C8	−178.3 (7)
N3—C4—C4A—C5	178.7 (6)	C7—C8—C8A—N1	178.8 (7)
C8A—C4A—C5—C6	−1.2 (11)	C7—C8—C8A—C4A	−1.2 (11)

Symmetry code: (i) −*x*+2, −*y*+1, −*z*.

### catena-Poly[[[quinazolin-4(3H)-one-κN3]mercury(II)]-di-µ-chlorido] (II) Hydrogen-bond geometry (Å, º)

**Table:**

*D*—H···*A*	*D*—H	H···*A*	*D*···*A*	*D*—H···*A*
N1—H1···O1^ii^	0.89 (4)	2.11 (4)	2.935 (8)	155 (7)

Symmetry code: (ii) *x*−1, *y*, *z*.

### Diiodidobis[quinazolin-4(3H)-one-κN3]cadmium(II) (III) Crystal data

**Table:**

[CdI_2_(C_16_H_12_N_4_O_2_)]	*F*(000) = 1224
*M**_r_* = 658.50	*D*_x_ = 2.453 Mg m^−^^3^
Monoclinic, *C*2/*c*	Mo *K*α radiation, λ = 0.71073 Å
*a* = 22.242 (3) Å	Cell parameters from 7294 reflections
*b* = 6.8450 (9) Å	θ = 3.1–30.6°
*c* = 13.3702 (17) Å	µ = 4.70 mm^−^^1^
β = 118.8220 (16)°	*T* = 100 K
*V* = 1783.4 (4) Å^3^	Plate, colourless
*Z* = 4	0.12 × 0.10 × 0.04 mm

### Diiodidobis[quinazolin-4(3H)-one-κN3]cadmium(II) (III) Data collection

**Table:**

Bruker D8 gonimeter with APEX CCD detector diffractometer	2687 independent reflections
Radiation source: Incoatec microsource	2519 reflections with *I* > 2σ(*I*)
Multilayer optics monochromator	*R*_int_ = 0.024
ω scans	θ_max_ = 30.7°, θ_min_ = 3.1°
Absorption correction: multi-scan (*SADABS*; Bruker, 2014)	*h* = −31→31
*T*_min_ = 0.544, *T*_max_ = 0.746	*k* = −9→9
13210 measured reflections	*l* = −18→18

### Diiodidobis[quinazolin-4(3H)-one-κN3]cadmium(II) (III) Refinement

**Table:**

Refinement on *F*^2^	Primary atom site location: structure-invariant direct methods
Least-squares matrix: full	Hydrogen site location: mixed
*R*[*F*^2^ > 2σ(*F*^2^)] = 0.026	H atoms treated by a mixture of independent and constrained refinement
*wR*(*F*^2^) = 0.063	*w* = 1/[σ^2^(*F*_o_^2^) + (0.0331*P*)^2^ + 1.7*P*] where *P* = (*F*_o_^2^ + 2*F*_c_^2^)/3
*S* = 1.12	(Δ/σ)_max_ = 0.001
2687 reflections	Δρ_max_ = 3.17 e Å^−^^3^
119 parameters	Δρ_min_ = −0.49 e Å^−^^3^
2 restraints	

### Diiodidobis[quinazolin-4(3H)-one-κN3]cadmium(II) (III) Special details

**Table:**

Geometry. All esds (except the esd in the dihedral angle between two l.s. planes) are estimated using the full covariance matrix. The cell esds are taken into account individually in the estimation of esds in distances, angles and torsion angles; correlations between esds in cell parameters are only used when they are defined by crystal symmetry. An approximate (isotropic) treatment of cell esds is used for estimating esds involving l.s. planes.

### Diiodidobis[quinazolin-4(3H)-one-κN3]cadmium(II) (III) Fractional atomic coordinates and isotropic or equivalent isotropic displacement parameters (Å^2^)

**Table:**

	*x*	*y*	*z*	*U*_iso_*/*U*_eq_	Occ. (<1)
I1	−0.02284 (2)	0.27011 (3)	0.06205 (2)	0.01738 (7)	
Cd1	0.000000	0.06009 (4)	0.250000	0.01209 (8)	0.9682 (8)
Cd2	0.000000	−0.5534 (10)	0.250000	0.01209 (8)	0.0318 (8)
O1	−0.15047 (11)	0.1341 (3)	0.16751 (18)	0.0192 (4)	
N1	−0.14386 (12)	−0.4477 (3)	0.2024 (2)	0.0156 (5)	
H1A	−0.1400 (18)	−0.583 (5)	0.200 (3)	0.019*	
C2	−0.08880 (14)	−0.3397 (4)	0.2268 (2)	0.0163 (5)	
H2A	−0.046045	−0.405307	0.254856	0.020*	
N3	−0.08910 (11)	−0.1482 (3)	0.21497 (19)	0.0135 (4)	
C4	−0.15069 (13)	−0.0452 (4)	0.1747 (2)	0.0117 (5)	
C4A	−0.21284 (13)	−0.1587 (4)	0.1429 (2)	0.0120 (5)	
C5	−0.27777 (14)	−0.0702 (4)	0.0976 (2)	0.0161 (5)	
H5A	−0.282158	0.066631	0.083981	0.019*	
C6	−0.33542 (15)	−0.1813 (5)	0.0726 (2)	0.0188 (6)	
H6A	−0.379070	−0.120238	0.042601	0.023*	
C7	−0.32946 (15)	−0.3836 (5)	0.0915 (2)	0.0191 (6)	
H7A	−0.369040	−0.458717	0.075289	0.023*	
C8	−0.26645 (15)	−0.4744 (4)	0.1334 (2)	0.0172 (5)	
H8A	−0.262633	−0.611862	0.144689	0.021*	
C8A	−0.20823 (13)	−0.3616 (4)	0.1592 (2)	0.0131 (5)	

### Diiodidobis[quinazolin-4(3H)-one-κN3]cadmium(II) (III) Atomic displacement parameters (Å^2^)

**Table:**

	*U*^11^	*U*^22^	*U*^33^	*U*^12^	*U*^13^	*U*^23^
I1	0.01805 (11)	0.01668 (11)	0.01536 (10)	−0.00240 (6)	0.00642 (8)	0.00158 (6)
Cd1	0.01073 (13)	0.00997 (13)	0.01466 (14)	0.000	0.00539 (11)	0.000
Cd2	0.01073 (13)	0.00997 (13)	0.01466 (14)	0.000	0.00539 (11)	0.000
O1	0.0209 (10)	0.0124 (10)	0.0246 (11)	−0.0019 (8)	0.0112 (9)	−0.0008 (8)
N1	0.0173 (11)	0.0099 (11)	0.0214 (12)	0.0013 (8)	0.0108 (10)	0.0023 (9)
C2	0.0129 (12)	0.0168 (13)	0.0194 (13)	0.0036 (10)	0.0080 (11)	0.0029 (11)
N3	0.0115 (10)	0.0147 (11)	0.0142 (10)	−0.0005 (8)	0.0060 (9)	0.0006 (9)
C4	0.0151 (12)	0.0102 (12)	0.0105 (11)	−0.0007 (9)	0.0067 (10)	−0.0012 (9)
C4A	0.0134 (12)	0.0124 (12)	0.0105 (11)	0.0001 (9)	0.0059 (10)	−0.0011 (9)
C5	0.0166 (12)	0.0156 (13)	0.0154 (13)	0.0020 (10)	0.0072 (11)	0.0001 (10)
C6	0.0124 (12)	0.0289 (16)	0.0140 (13)	0.0016 (11)	0.0055 (10)	−0.0019 (11)
C7	0.0165 (13)	0.0259 (15)	0.0159 (13)	−0.0066 (11)	0.0087 (11)	−0.0028 (11)
C8	0.0212 (14)	0.0156 (13)	0.0167 (13)	−0.0050 (11)	0.0108 (11)	−0.0026 (10)
C8A	0.0146 (12)	0.0136 (13)	0.0111 (11)	−0.0009 (10)	0.0061 (10)	−0.0005 (9)

### Diiodidobis[quinazolin-4(3H)-one-κN3]cadmium(II) (III) Geometric parameters (Å, º)

**Table:**

I1—Cd2^i^	2.608 (3)	N3—C4	1.397 (3)
I1—Cd1	2.7219 (4)	C4—C4A	1.459 (4)
Cd1—N3^ii^	2.299 (2)	C4A—C8A	1.402 (4)
Cd1—N3	2.299 (2)	C4A—C5	1.406 (4)
Cd2—C2^ii^	2.355 (5)	C5—C6	1.386 (4)
Cd2—C2	2.355 (5)	C5—H5A	0.9500
O1—C4	1.232 (3)	C6—C7	1.403 (5)
N1—C2	1.329 (4)	C6—H6A	0.9500
N1—C8A	1.391 (4)	C7—C8	1.381 (4)
N1—H1A	0.93 (4)	C7—H7A	0.9500
C2—N3	1.320 (4)	C8—C8A	1.401 (4)
C2—H2A	0.9500	C8—H8A	0.9500
			
N3^ii^—Cd1—N3	103.32 (12)	C2—N3—Cd1	129.61 (17)
N3^ii^—Cd1—I1	105.94 (6)	C4—N3—Cd1	110.74 (17)
N3—Cd1—I1	112.37 (6)	O1—C4—N3	119.5 (2)
N3^ii^—Cd1—I1^ii^	112.37 (6)	O1—C4—C4A	123.2 (2)
N3—Cd1—I1^ii^	105.94 (6)	N3—C4—C4A	117.3 (2)
I1—Cd1—I1^ii^	116.237 (16)	C8A—C4A—C5	118.5 (2)
C2^ii^—Cd2—C2	103.2 (3)	C8A—C4A—C4	119.6 (2)
C2^ii^—Cd2—I1^iii^	100.14 (7)	C5—C4A—C4	121.9 (2)
C2—Cd2—I1^iii^	113.53 (8)	C6—C5—C4A	120.4 (3)
C2^ii^—Cd2—I1^iv^	113.53 (8)	C6—C5—H5A	119.8
C2—Cd2—I1^iv^	100.14 (7)	C4A—C5—H5A	119.8
I1^iii^—Cd2—I1^iv^	124.8 (3)	C5—C6—C7	120.1 (3)
C2—Cd2—H2A^ii^	97.1 (4)	C5—C6—H6A	119.9
I1^iii^—Cd2—H2A^ii^	95.37 (11)	C7—C6—H6A	119.9
I1^iv^—Cd2—H2A^ii^	123.21 (12)	C8—C7—C6	120.5 (3)
C2—N1—C8A	120.6 (2)	C8—C7—H7A	119.8
C2—N1—H1A	118 (2)	C6—C7—H7A	119.8
C8A—N1—H1A	120 (2)	C7—C8—C8A	119.2 (3)
N3—C2—N1	124.9 (2)	C7—C8—H8A	120.4
N3—C2—Cd2	126.0 (2)	C8A—C8—H8A	120.4
N1—C2—Cd2	107.6 (2)	N1—C8A—C8	120.9 (3)
N3—C2—H2A	117.6	N1—C8A—C4A	117.9 (2)
N1—C2—H2A	117.6	C8—C8A—C4A	121.2 (2)
C2—N3—C4	119.6 (2)		
			
C8A—N1—C2—N3	1.1 (4)	C8A—C4A—C5—C6	1.7 (4)
C8A—N1—C2—Cd2	−165.5 (2)	C4—C4A—C5—C6	−177.4 (3)
N1—C2—N3—C4	0.4 (4)	C4A—C5—C6—C7	−0.6 (4)
Cd2—C2—N3—C4	164.55 (19)	C5—C6—C7—C8	−1.0 (4)
N1—C2—N3—Cd1	−179.0 (2)	C6—C7—C8—C8A	1.3 (4)
C2—N3—C4—O1	177.5 (2)	C2—N1—C8A—C8	−179.9 (3)
Cd1—N3—C4—O1	−3.0 (3)	C2—N1—C8A—C4A	−0.2 (4)
C2—N3—C4—C4A	−2.6 (4)	C7—C8—C8A—N1	179.5 (3)
Cd1—N3—C4—C4A	176.94 (17)	C7—C8—C8A—C4A	−0.1 (4)
O1—C4—C4A—C8A	−176.8 (2)	C5—C4A—C8A—N1	179.0 (2)
N3—C4—C4A—C8A	3.3 (3)	C4—C4A—C8A—N1	−1.9 (4)
O1—C4—C4A—C5	2.3 (4)	C5—C4A—C8A—C8	−1.4 (4)
N3—C4—C4A—C5	−177.6 (2)	C4—C4A—C8A—C8	177.7 (2)

Symmetry codes: (i) *x*, *y*+1, *z*; (ii) −*x*, *y*, −*z*+1/2; (iii) *x*, *y*−1, *z*; (iv) −*x*, *y*−1, −*z*+1/2.

### Diiodidobis[quinazolin-4(3H)-one-κN3]cadmium(II) (III) Hydrogen-bond geometry (Å, º)

**Table:**

*D*—H···*A*	*D*—H	H···*A*	*D*···*A*	*D*—H···*A*
N1—H1*A*···O1^iii^	0.93 (3)	1.97 (3)	2.893 (3)	168 (4)

Symmetry code: (iii) *x*, *y*−1, *z*.

## References

[bb1] Addison, A. W., Rao, T. N., Reedijk, J., van Rijn, J. & Verschoor, G. C. (1984). *J. Chem. Soc. Dalton Trans.* pp. 1349–1356.

[bb2] Bruker (2014). *APEX2*, *SAINT, *SADABS* * and *TWINABS*. Bruker AXS Inc., Madison, Wisconsin, USA.

[bb3] Đaković, M., Soldin, Ž., Kukovec, B.-M., Kodrin, I., Aakeröy, C. B., Baus, N. & Rinkovec, T. (2018). *IUCrJ*, **5**, 13–21. 10.1107/S2052252517015494 PMC5755573 29354267

[bb4] Groom, C. R., Bruno, I. J., Lightfoot, M. P. & Ward, S. C. (2016). *Acta Cryst.* B**72**, 171–179. 10.1107/S2052520616003954 PMC4822653 27048719

[bb5] Holmes, R. R. (1984). *Prog. Inorg. Chem.* **32**, 119–235.

[bb6] Hu, C. & Englert, U. (2001). *CrystEngComm*, **3**, 91–95.

[bb7] Hu, C. & Englert, U. (2002). *CrystEngComm*, **4**, 20–25.

[bb8] Hu, C., Kalf, I. & Englert, U. (2007). *CrystEngComm*, **9**, 603–610.

[bb9] Hu, C., Li, Q. & Englert, U. (2003). *CrystEngComm*, **5**, 519–529.

[bb10] Li, S. X., Liao, B. L., Luo, P. & Jiang, Y. M. (2015). *Chin. J. Inorg. Chem.* **31**, 291–296.

[bb11] Merkens, C., Kalf, I. & Englert, U. (2010). *Z. Anorg. Allg. Chem.* **636**, 681–684.

[bb12] Merkens, C., Truong, K.-N. & Englert, U. (2014). *Acta Cryst.* B**70**, 705–713. 10.1107/S2052520614006210 25080249

[bb13] Sheldrick, G. M. (2015*a*). *Acta Cryst.* A**71**, 3–8.

[bb14] Sheldrick, G. M. (2015*b*). *Acta Cryst.* C**71**, 3–8.

[bb15] Shomurotova, S., Turgunov, K. K., Mukhamedov, N. & Tashkhodjaev, B. (2012). *Acta Cryst.* E**68**, m724. 10.1107/S1600536812019381 PMC3379069 22719290

[bb16] Spek, A. L. (2009). *Acta Cryst.* D**65**, 148–155. 10.1107/S090744490804362X PMC2631630 19171970

[bb17] Spek, A. L. (2020). *Acta Cryst.* E**76**, 1–11. 10.1107/S2056989019016244 PMC6944088 31921444

[bb18] Terwingen, S. van, Nachtigall, N., Ebel, B. & Englert, U. (2021). *Cryst. Growth Des.* **21**, 2962–2969.

[bb19] Truong, K.-N., Merkens, C. & Englert, U. (2017). *Acta Cryst.* B**73**, 981–991. 10.1107/S2052520617011118 28981005

[bb20] Turgunov, K. & Englert, U. (2010). *Acta Cryst.* E**66**, m1457. 10.1107/S1600536810041590 PMC3009324 21588875

[bb21] Turgunov, K., Shomurotova, S., Mukhamedov, N. & Tashkhodjaev, B. (2010). *Acta Cryst.* E**66**, m1680. 10.1107/S1600536810048890 PMC3011606 21589337

[bb22] Turgunov, K. K., Wang, Y., Englert, U. & Shakhidoyatov, K. M. (2011). *Acta Cryst.* E**67**, m953–m954. 10.1107/S1600536811022471 PMC3151818 21836934

[bb23] Westrip, S. P. (2010). *J. Appl. Cryst.* **43**, 920–925.

[bb24] Yang, L., Powell, D. R. & Houser, R. P. (2007). *Dalton Trans.* pp. 955–964. 10.1039/b617136b 17308676

